# Complete Genome Sequence of *Bacillus velezensis* CN026 Exhibiting Antagonistic Activity against Gram-Negative Foodborne Pathogens

**DOI:** 10.1128/genomeA.01543-17

**Published:** 2018-01-25

**Authors:** Catherine Nannan, Annika Gillis, Simon Caulier, Jacques Mahillon

**Affiliations:** a Laboratory of Food and Environmental Microbiology, Earth and Life Institute, Université Catholique de Louvain, Louvain-la-Neuve, Belgium; b Phytopathology–Applied Microbiology, Earth and Life Institute, Université Catholique de Louvain, Louvain-la-Neuve, Belgium

## Abstract

We report here the complete genome sequence of *Bacillus velezensis* strain CN026, a member of the *B. subtilis* group, which is known for its many industrial applications. The genome contains 3,995,812 bp and displays six gene clusters potentially involved in strain CN026’s activity against Gram-negative foodborne pathogens.

## GENOME ANNOUNCEMENT

The *Bacillus subtilis* group originally comprised the species *B. subtilis*, *B. licheniformis*, *B. pumilus*, and *B. amyloliquefaciens* (1, 2). New species and subspecies were then validly described and added to this group (3, 4). In recent years, heterotypic synonyms between several species of the group have been highlighted, leading to taxonomic status reevaluations (4, 5). Hence, *B. amyloliquefaciens* subsp. *plantarum* (which includes the plant growth-promoting type strain FZB42 [6]), *B. methylotrophicus* (7), and *B. oryzicola* (8) have been suggested as later heterotypic synonyms of the species *B. velezensis* (9).

Strain CN026, isolated from chicken feces in Sorinnes, Belgium, was identified by *gyrB* and *rpoB* gene sequencing as belonging to the *B. velezensis* species (4, 10). Its culture supernatant displays a high ability to inhibit the growth of several important foodborne pathogens, i.e., *Escherichia coli*, *Salmonella enterica*, *Campylobacter jejuni*, *Listeria* spp., and *Bacillus* spp., in laboratory conditions, as well as in food matrices. Here, we report the complete genome sequence of strain CN026.

Whole-genome sequencing of strain CN026 was performed at Macrogen, Inc. (South Korea) using the Illumina MiSeq platform with a 300-bp paired-end run and 550-bp insert library (TruSeq DNA PCR-Free). A total of 1,888,971 reads were generated and assembled in 28 contigs with the IDBA-UD *de novo* assembler (11). The contigs were ordered and oriented using CAR analysis (12) based on the reference genome of the closely related *B. velezensis* strain JS25R (GenBank accession number NZ_CP009679.1). The gaps between contigs were filled by custom primer walking, PCR amplification, and DNA sequencing. Gene annotation was performed with the NCBI Prokaryotic Genome Annotation Pipeline (PGAP) version 4.3 (13).

The *B. velezensis* strain CN026 genome contains a single circular chromosome of 3,995,812 bp with a G+C content of 46.4%. It comprises 3,875 coding sequences and 68 tRNAs. A genome-to-genome distance comparison with strain JS25R was calculated *in silico* (14). The DNA-DNA hybridization was estimated to be 93.60%, whereas the evaluated average nucleotide identity was 99.24% (15). No complete prophage region and no acquired antimicrobial resistance gene were identified with the PHAST (16) and ResFinder (17) search tools, respectively. Similarly, no gene families that correlate with human pathogenicity were detected in the CN026 genome (18).

AntiSMASH analyses were performed to identify gene clusters encoding the biosynthetic pathways of known classes of secondary metabolites (19). Six complete gene cluster sequences were detected: three polyketide synthases for macrolactin, bacillaene, and difficidin, as well as three nonribosomal peptide synthetases for fengycin, bacillibactin, and bacilysin. Overall, strain CN026 represents a good candidate for use in biopreservation.

### Accession number(s).

The complete genome sequence of strain CN026 was deposited in GenBank under the accession no. CP024897.
